# Gypenoside XLIX and Mitochondria-Associated ER Membranes in Non-Alcoholic Fatty Liver Disease: Mechanistic Insights and Emerging Perspectives

**DOI:** 10.3390/molecules31081325

**Published:** 2026-04-17

**Authors:** Xinyi Kwan, Muhammad Shahzad Aslam, Huiqing Liang, Shaodong Chen

**Affiliations:** 1 Department of Traditional Chinese Medicine, School of Medicine, Xiamen University, Xiamen 361102, China; kwanxinyi0808@gmail.com (X.K.); aslam.shahzad@xmu.edu.my (M.S.A.); 2 School of Traditional Chinese Medicine, Xiamen University Malaysia, Sepang 43900, Malaysia; 3 Hepatology Unit, Xiamen Hospital of Traditional Chinese Medicine, Xiamen 361009, China

**Keywords:** Gypenoside XLIX, non-alcoholic fatty liver disease, mitochondria-associated endoplasmic reticulum membranes, mitochondrial dysfunction, calcium homeostasis

## Abstract

Gypenoside XLIX is a bioactive saponin with reported diverse biological activities, including antioxidant, regulation of cell growth, immune responses, and metabolic regulatory properties. The increasing global prevalence of non-alcoholic fatty liver disease (NAFLD) underscores the importance of exploring novel therapeutic agents such as Gypenoside XLIX. NAFLD pathogenesis involves lipotoxicity, oxidative stress, and mitochondrial dysfunction, in which mitochondria-associated endoplasmic reticulum membranes (MAMs) play a critical role in organelle communication, calcium signaling, and lipid metabolism. This narrative review summarizes current evidence indicating that Gypenoside XLIX may modulate oxidative stress, restore mitochondrial membrane potential, and regulate calcium homeostasis, thereby indirectly influencing MAM integrity and function. These effects can reduce lipid accumulation, improve hepatocellular metabolism, and attenuate inflammatory responses. This review evaluates the mechanistic impact and function of Gypenoside XLIX on MAM integrity and its effects on NAFLD. However, there is limited direct experimental evidence linking Gypenoside XLIX to MAM regulation, and further studies are required to validate its mechanisms and therapeutic potential in clinical settings.

## 1. Introduction

Non-alcoholic fatty liver disease (NAFLD), recently redefined as metabolic dysfunction-associated steatotic liver disease (MASLD) [1], is a chronic liver disease characterized by abnormal fat accumulation in liver cells. Its global prevalence is showing a significant upward trend and has become the leading cause of chronic liver disease [2]. The characteristics of the affected population indicate that obesity, type 2 diabetes, and metabolic syndrome are important risk factors for NAFLD. The prevalence of overweight or obese people is significantly higher than that of people with normal weight [3]. NAFLD not only affects liver function, but may also further develop into non-alcoholic steatohepatitis (NASH), cirrhosis, or even hepatocellular carcinoma (HCC), posing a serious threat to the health of patients [4,5]. Therefore, in-depth research on the pathogenesis of NAFLD and its treatment strategies has important clinical significance.

Mitochondria-associated endoplasmic reticulum membranes (MAMs) have emerged as critical regulators of cellular homeostasis, facilitating communication between the endoplasmic reticulum and mitochondria as well as lipid metabolism and mitochondrial function, all of which are closely linked to the pathogenesis of NAFLD [6,7]. Dysregulation of MAMs has been increasingly recognized as a contributing factor to metabolic liver disease progression.

*Gynostemma pentaphyllum* (GP) and its major active component, gypenosides, have been extensively studied for their hepatoprotective effects. Among them, Gypenoside XLIX, a bioactive saponin isolated from Gynostemma pentaphyllum, has attracted attention due to its antioxidant, anti-inflammatory, and metabolic regulatory effects. Recent studies have shown that Gypenoside XLIX may influence mitochondrial function, oxidative stress, and calcium homeostasis. This suggests it likely playing a role in MAM-associated pathways in NAFLD [8,9].

However, no review has systematically explored the potential link between Gypenoside XLIX and MAM regulation in NAFLD. Therefore, this review aims to summarize current evidence on the biological effects of Gypenoside XLIX and to explore its potential role in modulating MAM-associated mechanisms in the context of NAFLD.

## 2. Literature Search Strategy

A non-systematic review of English-language literature was conducted by searching major scientific databases, including Web of Science, Scopus, EMBASE, and PubMed. The search was performed using combinations of the following keywords: “Gypenoside XLIX”, “Gynostemma pentaphyllum”, “non-alcoholic fatty liver disease”, “mitochondria-associated ER membranes”, “calcium signaling”, and “oxidative stress”. This review included both in vitro and in vivo studies published up to 2025 and was selected based on their relevant studies on investigating the biological activities of Gypenoside XLIX, particularly its effects on mitochondrial function, oxidative stress, calcium homeostasis, and MAM-related signaling pathways in the context of NAFLD.

We included peer-reviewed, animal, and clinical studies that investigated the biological effects of Gypenoside XLIX, particularly in the context of liver disease, metabolic regulation, and mitochondrial or endoplasmic reticulum-related functions. Priority was given to studies exploring mechanisms of action, including those involving oxidative stress, lipid metabolism, calcium homeostasis, and inter-organelle communication.

We excluded studies that did not focus on Gypenoside XLIX, as well as studies focusing solely on NAFLD without evaluating its effects. Non-English publications and studies addressing general gypenosides without specific reference to Gypenoside XLIX were also excluded.

## 3. Pathogenesis of NAFLD and Role of MAMs

### 3.1. FFA-Induced Injury

Free fatty acids (FFAs), typically represented by a mixture of oleic acid and palmitic acid, are commonly used to establish lipid models in NAFLD cell experiments [10,11]. The cellular injury induced by FFAs is not due to the molecules themselves, but rather to the metabolic stress caused by their excessive accumulation. Under such conditions, FFAs are stored in lipid droplets, leading to increased triglyceride (TG) deposition [12].

### 3.2. Mitochondrial Dysfunction

Excess free fatty acids and lipid accumulation in hepatocytes overwhelms the mitochondrial β-oxidation pathway, resulting in reduced adenosine triphosphate (ATP) synthesis and the accumulation of reactive oxygen species (ROS) and acylcarnitine [13,14]. Elevated ROS levels disrupt organelle function, fragment mitochondria, and compress the endoplasmic reticulum (ER), causing deformation of its morphology. This is accompanied by disturbances in calcium homeostasis and impaired protein folding, ultimately triggering ER stress [13,15,16]. In parallel, lysosomal rupture, decreased mitochondrial membrane potential, and diminished ATP production further compromise hepatocyte function [17,18].

As a consequence, hepatocytes shift from oxidative metabolism to a lipotoxic state, initiating the pathological cascade that progresses from steatosis to steatohepatitis and eventually fibrosis in NAFLD. To comprehensively evaluate these pathological changes, a series of biochemical and fluorescence-based assays is commonly employed in in vitro models.

### 3.3. Experimental Readouts

Oil Red O staining and intracellular TG quantification are widely used to assess lipid accumulation and steatosis in hepatocytes [19,20], providing direct evidence of FFAs-induced lipid deposition. Excessive lipid accumulation subsequently promotes oxidative stress, which is reflected by increased intracellular ROS levels [18]. Therefore, ROS detection using dichlorodihydrofluorescein diacetate (DCFH-DA) serves as a key indicator of oxidative injury.

Mitochondria are primary targets of lipotoxic damage. Disruption of mitochondrial membrane potential (ΔΨm) represents an early event in mitochondrial dysfunction and can be effectively evaluated using the JC-1 fluorescent probe. In addition, impaired mitochondrial function leads to reduced ATP production, indicating compromised cellular energy metabolism [21].

Collectively, the combined assessment of lipid accumulation (Oil Red O and TG), oxidative stress (ROS), mitochondrial membrane potential (JC-1), and cellular energy status (ATP) allows for a systematic evaluation of FFAs-induced lipotoxicity and the protective effects of therapeutic interventions in HepG2 cells. These events collectively establish lipotoxicity-induced mitochondrial dysfunction as a central driver of NAFLD progression, providing a mechanistic basis for targeting mitochondrial stress responses and organelle communication.

### 3.4. Structure and Core Proteins of MAMs

Mitochondria-Associated Endoplasmic Reticulum Membranes (MAMs) are intercellular communication platforms formed by the connection between the ER and mitochondria through specific membrane structures. They are complex and dynamic, containing a variety of protein and lipid components. MAMs play a key role in cell metabolism, calcium signaling, lipid transport and apoptosis, especially in regulating mitochondrial function [22,23]. Naon and Scorrano provided one of the most authoritative descriptions of ER-mitochondrial contact, defining MAMs as dynamic microregions maintained at a precise distance of 10–50 nm. They emphasized the importance of protein complexes such as IP3R-Grp75-VDAC1 and MFN2 in coordinating Ca^2+^ transport and metabolic communication, providing a structural basis for understanding the biological characteristics of MAMs in liver diseases [24]. In recent years, MAMs have become potential therapeutic targets due to their extensive involvement in the pathophysiological processes of various diseases. For example, in metabolic diseases, dysfunction of MAMs can lead to mitochondrial dysfunction and lipid metabolism disorders, thereby promoting the occurrence and development of NAFLD. Furthermore, MAMs are involved in regulating inflammatory responses and oxidative stress, which play a crucial role in the progression of NAFLD. Therefore, research on MAMs not only helps to elucidate the mechanisms of disease development but also provides new directions for developing novel therapeutic strategies.

### 3.5. MAMs and Ca^2+^ Transport

MAMs play a crucial role in calcium ion transport between the ER and mitochondria. Through MAMs, calcium released from the ER enters mitochondria via the mitochondrial calcium uniporter, causing oxidant stress [25]. The function of MAMs has mainly been associated with calcium transfer, lipid synthesis, autophagy, and ROS [26]. In pathological conditions of oxidative stress, excess mitochondrial ROS can stimulate calcium release from the ER. Elevated mitochondrial Ca^2+^ triggers mitochondrial membrane depolarization (loss of ΔΨm), indicating disrupted mitochondrial function [27,28]. Calcium overloading caused ROS generation in mitochondria which contributes to membrane potential changes and mitochondrial damages [29]. This ROS–ER–Ca^2+^–mitochondria loop has been shown to drive programmed cell death pathways and highlights the interdependent relationship between oxidative stress and mitochondrial calcium handling in cell injury models.

### 3.6. MAMs and Lipid Metabolism

MAMs play a central role in coordinating lipid metabolism [30], linking the ER and mitochondria. At membrane contact sites, MAMs coordinate lipid transport between organelles, supporting phospholipid synthesis and transport, lipid droplet formation, cholesterol esterification [31], fatty acid metabolism, calcium homeostasis, and mitochondrial dynamics. Lipid metabolism involving phospholipids, sphingolipids, and non-mitochondrial fatty acids is closely associated with organelle interactions at these sites, where lipids are exchanged between the ER and mitochondria [32,33]. When MAM function becomes dysregulated, these processes shift toward fatty degeneration [34], leading to excessive triglyceride accumulation in hepatocytes and the development of steatosis characteristic of NAFLD.

### 3.7. Evidence for Dysregulated MAMs in NAFLD

**Figure 1:**
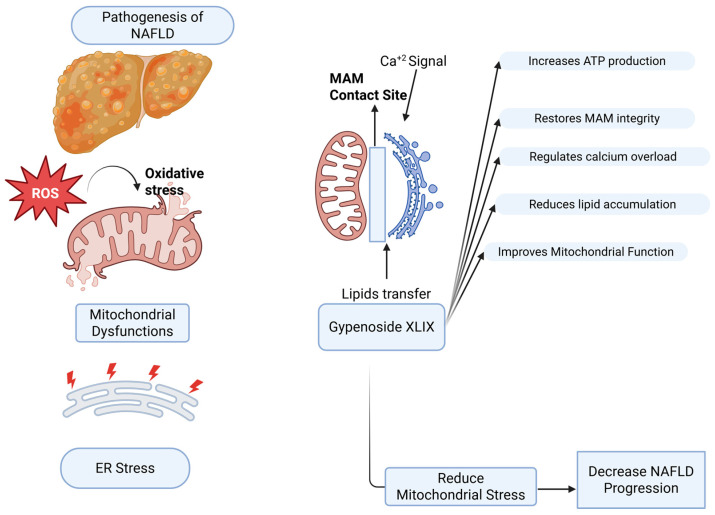
Proposed mechanism of Gypenoside XLIX in mitigating NAFLD progression via modulation of mitochondria-associated ER membranes (MAMs). In NAFLD, lipotoxicity induces oxidative stress, mitochondrial dysfunction, ER stress and impaired organelle communication. MAMs disruption leads to altered calcium signaling, lipid transfer imbalance and reduced mitochondrial ATP production. Gypenoside XLIX may restore MAMs’ integrity, improve mitochondrial function, regulate calcium homeostasis and attenuate lipid accumulation, thereby slowing NAFLD progression. The illustration was created using BioRender.com.

In NAFLD, the liver develops mitochondrial dysfunction [35], accompanied by altered physical communication between the ER and mitochondria. These contact sites, known as MAMs, regulate calcium transfer from the ER to mitochondria. When MAMs activity becomes dysregulated, calcium flux is disturbed, contributing to mitochondrial dysfunction and disease progression [36,37]. Excessive MAMs activity during ER stress can drive calcium overload in mitochondria, leading to loss of membrane potential, increased ROS, and oxidative stress [38,39,40]. Conversely, weakened ER–mitochondria contacts reduce calcium influx, impair ATP production, and disrupt metabolic homeostasis [41]. Thus, both hyperactivation and loss of MAMs function contribute to steatosis, organelle stress, and hepatocyte injury, underscoring their role as potential therapeutic targets. Importantly, MAMs dysfunction in NAFLD should be understood not as a simple increase or decrease in activity, but as a disturbance of dynamic homeostasis that destabilizes calcium signaling and mitochondrial integrity [40,41,42]. Given the central role of MAMs in coordinating calcium signaling, lipid metabolism, and mitochondrial function, compounds capable of modulating these interconnected pathways may represent promising therapeutic candidates for NAFLD. To illustrate the proposed role of Gypenoside XLIX in modulating MAM-associated pathways in NAFLD, a schematic overview is presented in Figure 1.

## 4. Biological Effects of Gypenoside XLIX

Evidence indicates that gypenosides ameliorate NAFLD and metabolic disorders by regulating lipid metabolism by suppressing lipogenesis and promoting fatty acid oxidation; enhancing antioxidant defenses by reducing intracellular ROS, and alleviating oxidative stress–induced cellular injury. In addition, gypenosides activate key metabolic pathways, including AMPK/SIRT1/PGC-1α, thereby promoting mitochondrial biogenesis and maintaining cellular energy homeostasis [43,44,45]. Their protective actions involve multiple mechanisms, including modulation of lipid metabolic enzymes, activation of AMPK, anti-inflammatory effects [9,43,46,47], and reinforcement of cellular antioxidant capacity.

Among the various bioactive gypenosides isolated from GP, Gypenoside XLIX (XLIX) was selected for further investigation based on its stability structural characteristics, reported biological activities, and preliminary experimental evidence [48]. Computational studies indicate that XLIX can form structurally stable complexes with potential protein targets [49]. Structurally, it is a dammarane-type triterpenoid saponin, closely related to ginsenosides [48], which are widely associated with lipid metabolism regulation, antioxidant activity, mitochondrial protection, and hepatoprotection [50].

Bioassays revealed that XLIX is the major component purified from the most active fraction of GP extract, with diverse pharmacological activities including lipid metabolism regulation, anti-atherosclerotic, anticancer, anti-inflammatory, antidiabetic, and anti-NAFLD effects [8]. In our preliminary screening, XLIX at 20 μM showed no cytotoxicity and exhibited protective tendencies under FFAs-induced lipotoxic conditions, supporting its selection as the target compound for mechanistic studies.

According to pharmacokinetic studies, XLIX has a very short half-life and extremely low oral bioavailability (~0.14%), likely due to its poor solubility, large molecular size, and rapid metabolism after administration in rats [49]. This limitation underscores the need for mechanistic in vitro studies and improved delivery strategies, such as nanoparticle formulations or structural modifications. Pharmacokinetic limitations of Gypenoside XLIX, including low oral bioavailability and rapid metabolism, may restrict its clinical application. Strategies such as nanoparticle-based delivery systems, structural modification, or formulation optimization may enhance its therapeutic potential.

## 5. Mechanistic Pathways Linking XLIX to NAFLD

### 5.1. Hepatic Injury and Metabolic Relevance

Transcriptomic analyses (GO and KEGG) demonstrated that XLIX significantly alters gene expression, enriching pathways related to glycerophospholipid metabolism, bile secretion, fatty acid degradation, ion transmembrane transport, and transcription factor activity [51]. In drug-induced liver injury models, XLIX reduced oxidative stress and inflammation via modulation of the Akt/NLRP3 inflammasome pathway [50]. Mechanistically, it inhibits NF-κB signaling to suppress pro-inflammatory responses, activates PPAR-α to enhance lipid metabolism and anti-inflammatory activity, and suppresses NLRP3 inflammasome activation to reduce pyroptosis and chronic inflammation [9]. As a PPAR-α activator, XLIX facilitates hepatic fatty acid processing and additionally inhibits tissue factor, a pro-thrombotic mediator linked to fibrosis and cirrhosis progression, thereby reducing the clot-promoting environment in chronic liver injury [8]. Although direct clinical studies on XLIX are limited, human trials using GP extract in NAFLD patients show improved markers of liver injury, insulin resistance, and metabolic parameters [52]. These hepatic findings are complemented by evidence of XLIX’s protective effects in extra-hepatic systems.

### 5.2. Extra-Hepatic Evidence

Beyond the liver, XLIX has demonstrated protective effects in multiple organ injury models. In sepsis-induced intestinal injury, it enhanced antioxidant enzyme levels, reduced ROS accumulation via Nrf2-Keap1 activation, and inhibited NLRP3 inflammasome activity. In sepsis-induced lung injury, it reduced inflammation and oxidative stress through Sirt1/Nrf2 signaling, while suppressing mitochondrial apoptosis and excessive autophagy via the Pink1/Parkin pathway. In ischemic stroke models, XLIX improved neuronal survival by activating PI3K/AKT signaling, reducing apoptosis and ROS accumulation, and silencing FOXO1 to restore mitochondrial membrane potential and optimize PINK1-mediated mitophagy [53,54,55]. These systemic effects reinforce XLIX’s role as a regulator of mitochondrial and inflammatory pathways across multiple tissues.

### 5.3. Preclinical and Clinical Evidence

Pre-clinical and clinical studies further demonstrate that gypenosides significantly reduce liver injury markers such as alanine aminotransferase (ALT), aspartate aminotransferase (AST) and alkaline phosphatase (ALP), improve blood lipid profiles by lowering triglycerides, total cholesterol, and low-density lipoprotein (LDL), and ameliorate liver histology by decreasing lipid vacuole size and reducing fibrosis or stiffness [46,56,57]. Moreover, clinical data suggest that GP extract used alongside dietary control is more effective in reducing BMI and hepatic fat accumulation than diet alone, highlighting its potential as a valuable adjunct therapy for metabolic liver diseases [58]. The anti-lipid deposition, antioxidant, and mitochondrial protective effects of gypenosides collectively constitute protective mechanism against metabolic liver diseases such as NAFLD. To provide a structured overview of the current experimental evidence supporting Gypenoside XLIX, the key findings from relevant studies are summarized in Table 1.

## 6. Limitations and Future Perspectives

### 6.1. Lack of Direct Evidence on MAM Regulation

As shown in Table 1, most studies provide indirect evidence across different biological systems, with limited direct investigation of MAM-related mechanisms. Collectively, these findings highlight that XLIX modulates multiple signaling pathways, including PI3K/AKT, Sirt1/Nrf2, PPAR-α, and NLRP3, that converge on mitochondrial function, calcium homeostasis, and lipid metabolism. These processes are tightly regulated at MAMs, suggesting a potential link between XLIX and MAMs dynamics.

Previous studies have suggested that gypenosides may play a role by regulating mitochondrial function and calcium signaling [63], but whether XLIX directly affects MAMs, especially ER-mitochondrial calcium transport mediated by key proteins such as VDAC1–IP3R1 [64], has not been systematically studied.

Recent mechanistic studies have highlighted that acetylation of the mitochondrial chaperone GRP75 disrupts ER–mitochondrial calcium transfer, impairs calcium homeostasis, and drives hepatocyte insulin resistance. These findings underscore the importance of VDAC1–IP3R1–GRP75 complexes in maintaining metabolic balance [65]. Although accumulating evidence indicates that XLIX modulates mitochondrial function and inflammatory signaling, there is currently no direct experimental validation of its effects on MAMs integrity or ER–mitochondrial calcium dynamics, nor incorporation of functional indicators such as lipid deposition or ROS.

### 6.2. Limited Evidence of XLIX In Vitro Lipotoxicity Models

Although there are numerous reports on the effects of total saponins from GP or related ginsenosides on lipid metabolism, antioxidation, and mitochondrial protection, research on specific monomers remains limited. In particular, while XLIX exhibits structural stability and significant activity, direct evidence for its use in in vitro lipotoxicity models is insufficient. Currently, there is limited evidence regarding the direct role of XLIX in FFAs-induced hepatocellular lipotoxicity models, and systematic validation is lacking [66].

### 6.3. Lack of Cascaded Evidence Chain

Existing experiments mostly involve observation of single indicators and failing to establish a complete chain of evidence. Previous studies have shown that total saponins from GP detection of lipid deposition and oxidative stress using Oil Red O and ROS, but without incorporating more indicators [43,67]. These studies lack a comprehensive assessment of mitochondrial function (JC-1, ATP, and Ca^2+^ dynamics) and MAMs regulation. Although XLIX has been reported to have anti-inflammatory, antioxidant, and mitochondrial protective effects in various models of kidney injury, liver injury, intestinal inflammation, stroke, and lung injury, involving signaling pathways such as NF-κB, PPAR-α, NLRP3, PI3K/AKT, and Sirt1/Nrf2 [8,50,54,55,60], these studies are mostly based on single models and single indicators, lacking evidence chain from multiple dimensions to comprehensively reveal the mechanism of action of XLIX in lipid metabolism, oxidative stress, mitochondrial function, and MAMs dynamics. Therefore, the role of XLIX in NAFLD-related lipotoxicity models still requires further systematic investigation.

Although gypenosides have been shown to induce apoptosis via sustained intracellular Ca^2+^ elevation in hepatoma cells [68], but their regulatory role in physiological ER-mitochondrial calcium homeostasis in metabolic hepatocytes remains unclear. These studies also did not include lipid metabolism assays (such as Oil Red O or triglyceride measurements), focusing primarily on Ca^2+^ overload and apoptosis. On the other hand, in NAFLD studies, ATP measurement is considered a key indicator reflecting mitochondrial function and energy metabolism status, but many studies use only a single indicator (such as ATP or ROS), failing to form a complete chain of evidence [69]. The JC-1 fluorescent probe is also widely used to detect mitochondrial membrane potential (ΔΨm), but most studies use this indicator alone without combining it with ATP or Ca^2+^ measurements [70].

### 6.4. Limitations of the HepG2 and FFA-Induced In Vitro Model

Although HepG2 and free fatty acid (FFA)-induced models are widely used to investigate NAFLD-related mechanisms, several limitations should be considered when interpreting these findings. HepG2 cells are a well-established human hepatic cell line widely used across many research areas. Their stable genetic background, good experimental reproducibility, and broad applicability in drug metabolism and toxicity screening make them a valuable tool [71]. They are routinely employed in the screening of hepatotoxic compounds and in evaluating drug metabolism. Although their metabolic profile only partially resembles that of normal liver cells, HepG2 cells can mimic certain hepatic functions, which is particularly useful for testing compounds targeting liver cancer or fibrosis. They also show high sensitivity to drug-induced liver injury and are frequently used in the initial screening of toxicity [72,73]. In addition, HepG2 cells can effectively take up and store exogenous fatty acids and residual lipoprotein particles, making them suitable for lipid metabolism studies [73]. Their unlimited lifespan, phenotypic stability, and reproducibility further support their widespread use in in vitro toxicity testing [71,74].

To induce hepatocyte steatosis, oleic acid (OA), palmitic acid (PA), or an OA/PA mixture is commonly applied, with NAFLD cell models typically using a FFAs mixture of OA and PA at a 2:1 molar ratio conjugated to bovine serum albumin (BSA) [75,76,77].

Despite these advantages, HepG2 cells have notable limitations. Compared with primary hepatocytes, they exhibit significant differences in metabolic enzyme profiles [71,78], particularly the under-expression of cytochrome P450 (CYP) enzymes [79]. Their low drug-metabolizing capacity and tendency toward rapid dedifferentiation during long-term culture result in transcriptional profiles that diverge from normal human hepatocytes. These differences limit their accuracy in simulating liver metabolism, so results must be interpreted cautiously in pharmacokinetic and toxicological studies and are often validated with additional models. Nevertheless, in NAFLD research, HepG2 cells remain a widely used in vitro system, with numerous studies highlighting their value in lipid metabolism, drug development, and toxicity evaluation [73].

### 6.5. Future Research Directions

However, these studies lack a multi-dimensional cascaded chain of evidence and have not yet systematically integrated comprehensive verification of lipid metabolism, oxidative stress, mitochondrial function, and MAMs dynamics.

Therefore, it is necessary to establish an FFAs-induced hepatocellular lipotoxicity model to validate the protective effect of XLIX. Within this system, multiple indicators should be assessed in parallel, including lipid deposition (Oil Red O, TG), oxidative stress (ROS), and mitochondrial function (ΔΨm, ATP) in the same experimental system, combined with mechanistic level techniques such as PLA (protein interaction), colocalization imaging, and Ca^2+^ flow assays, to construct a function–structure–signal transduction evidence chain that corroborates each other, reveal the mechanism of action of XLIX in NAFLD lipotoxicity. Through this comprehensive study, this integrated approach will enable the construction of a function–structure–signal transduction evidence chain, thereby revealing the mechanism of action of XLIX in NAFLD-related lipotoxicity.

## 7. Conclusions

Gypenoside XLIX has demonstrated antioxidant, anti-inflammatory, and mitochondrial protective effects, and has been shown to modulate key pathways involved in NAFLD pathogenesis, including PPAR-α, NLRP3, PI3K/AKT, and Sirt1/Nrf2. Through these mechanisms, it may regulate lipid metabolism, oxidative stress, and calcium homeostasis, processes that are closely associated with mitochondria-associated endoplasmic reticulum membranes (MAMs), suggesting a potential role in MAM regulation.

However, current evidence remains largely indirect, and the effects of Gypenoside XLIX on MAM structure and function have not been directly validated. In addition, most available studies are based on non-NAFLD or extra-hepatic models, limiting their relevance to metabolic liver disease. Future research should focus on validating these mechanisms using NAFLD-specific models and integrated experimental approaches that assess lipid accumulation, oxidative stress, mitochondrial function, and MAM dynamics to establish a comprehensive evidence framework.

Furthermore, pharmacokinetic limitations, such as low bioavailability and short half-life, highlight the need for improved delivery strategies. Overall, Gypenoside XLIX represents a promising candidate for further investigation in NAFLD therapy, particularly in the context of MAM-targeted interventions.

## Figures and Tables

**Table 1:** Mechanistic pathways of Gypenoside XLIX and relevance to NAFLD.

Study	Model	System	Key Findings	Mechanism/Pathway	Relevance to NAFLD	Relevance to MAM	Evidence Level
[51]	Fatty liver cells	Hepatic	Altered gene expression, lipid metabolism	KEGG: FA degradation, bile secretion	Regulates lipid metabolism and gene expression, suggesting potential to reduce hepatic steatosis and metabolic dysregulation in NAFLD	Potential	Indirect
[53]	CLP-induced intestinal injury	Intestinal	Reduced ROS and inflammation, increased Nrf2/Keap1 signaling, enhanced antioxidant enzyme activity, and improved barrier function	Nrf2/Keap1, NF-κB, PI3K/AKT	Inhibits NLRP3-mediated inflammation and reduces oxidative stress, suggesting potential to alleviate mitochondrial dysfunction and inflammatory progression in NAFLD	Indirect	Indirect
[9]	CLP-induced liver injury + RAW264.7	Hepatic	Reduced ALT and AST levels, decreased lipid accumulation and inflammatory cytokine expression, and increased antioxidant activity	NF-κB/PPAR-α/NLRP3 pathways	Inhibits inflammatory signaling and lipid accumulation, suggesting improvement of steatosis and hepatic inflammation in NAFLD	Potential	Indirect
[54]	MCAO + OGD neurons	Neural	Reduced ROS levels and apoptosis, improved mitochondrial function, and decreased infarct size	PI3K/AKT/FOXO1, mitophagy	Regulates mitochondrial autophagy and reduces ROS, suggesting potential to restore mitochondrial dysfunction and lipid metabolism imbalance in NAFLD	Indirect	Indirect
[8]	THP-1 monocytes	Immune	Reduced tissue factor expression	PPAR-α dependent	None	Not direct	Indirect
[59]	HUVEC cells	Vascular	Reduced adhesion molecule expression	PPAR-α pathway	None	Not direct	Indirect
[55]	CLP-induced ALI + MLE-12	Pulmonary	Reduced inflammatory cytokine levels, decreased apoptosis and ROS levels, and enhanced antioxidant response	Sirt1/Nrf2, Pink1/Parkin	Activates antioxidant pathways (Nrf2), reducing oxidative stress and inflammation, key drivers of NAFLD progression	Indirect	Indirect
[60]	CLP-induced encephalopathy	Neural	Reduced apoptosis and inflammation	PPAR-α activation	Involved pathways overlap with key mechanisms underlying NAFLD through modulation of PPAR-α, NF-κB, and NLRP3 signaling pathways	Indirect	Indirect
[61]	Mouse + tubular epithelial cells	Renal	Reduced renal injury markers, decreased inflammation, and decreased programmed cell death	IGFBP7/IGF1R; mitochondrial apoptosis	Modulates cell survival and metabolic signaling, potentially improving hepatocyte injury and insulin resistance in NAFLD	Indirect	Indirect
[50]	Diclofenac-induced liver injury (rat + L02 liver cells)	Hepatic	Reduced ALT, AST, and ALP levels, decreased ROS and inflammatory cytokines, and increased antioxidant enzyme activity	AKT/NLRP3 pathway	Inhibits inflammasome activation and ROS, suggesting mitigation of mitochondrial dysfunction and inflammatory progression in NAFLD	Potential	Indirect
[62]	UUO mouse + HK2 cells	Renal	Reduced collagen deposition, decreased fibrosis markers, and reduced Smad3 activation	TGF-β/Smad3 signaling	Regulates fibrosis pathways, potentially relevant to progression from NAFLD to liver fibrosis	Indirect	Indirect

## Data Availability

The authors have nothing to report. All data analyzed in the current review are extracted from published articles in PubMed, Web of Science, Scopus and EMBASE.

## Abbreviations

The following abbreviations are used in this manuscript:

ATP	Adenosine Triphosphate
ALT	Alanine Aminotransferase
ALP	Alkaline Phosphatase
AST	Aspartate Aminotransferase
BSA	Bovine Serum Albumin
CLP	Cecal Ligation and Puncture
CYP	Cytochrome P450
DCFH-DA	Dichlorodihydrofluorescein Diacetate
ER	Endoplasmic Reticulum
FFAs	Free Fatty Acids
GP	Gynostemma pentaphyllum
GO	Gene Ontology
JC-1	5,5′,6,6′-Tetrachloro-1,1′,3,3′-tetraethylbenzimidazolylcarbocyanine iodide
KEGG	Kyoto Encyclopedia of Genes and Genomes
XLIX	Gypenoside XLIX
HCC	Hepatocellular Carcinoma
LDL	Low-Density Lipoprotein
MCAO	Middle Cerebral Artery Occlusion
MAMs	Mitochondria-Associated Endoplasmic Reticulum Membranes
MASLD	Metabolic Dysfunction-Associated Steatotic Liver Disease
NAFLD	Non-Alcoholic Fatty Liver Disease
NASH	Non-Alcoholic Steatohepatitis
OA	Oleic Acid
OGD	Oxygen-Glucose Deprivation
PA	Palmitic Acid
PLA	Proximity Ligation Assay
ROS	Reactive Oxygen Species
TG	Triglyceride
